# 

**Published:** 1852-09

**Authors:** 


☞ A communication from Dr. Rogers, received too late for insertion in this No., will appear in our next issue.

